# Hsp40 Couples with the CSPα Chaperone Complex upon Induction of the Heat Shock Response

**DOI:** 10.1371/journal.pone.0004595

**Published:** 2009-02-26

**Authors:** Sarah J. Gibbs, Brandy Barren, Katy E. Beck, Juliane Proft, Xiaoxi Zhao, Tatiana Noskova, Andrew P. Braun, Nikolai O. Artemyev, Janice E. A. Braun

**Affiliations:** 1 Department of Physiology and Biophysics & Hotchkiss Brain Institute, University of Calgary, Calgary, Alberta, Canada; 2 Department of Pharmacology and Therapeutics & Libin Cardiovascular Institute of Alberta, University of Calgary, Calgary, Alberta, Canada; 3 Department of Molecular Physiology and Biophysics, University of Iowa, Iowa City, Iowa, United States of America; University of Cambridge, United Kingdom

## Abstract

In response to a conditioning stress, the expression of a set of molecular chaperones called heat shock proteins is increased. In neurons, stress-induced and constitutively expressed molecular chaperones protect against damage induced by ischemia and neurodegenerative diseases, however the molecular basis of this protection is not known. Here we have investigated the crosstalk between stress-induced chaperones and cysteine string protein (CSPα). CSPα is a constitutively expressed synaptic vesicle protein bearing a J domain and a cysteine rich “string” region that has been implicated in the long term functional integrity of synaptic transmission and the defense against neurodegeneration. We have shown previously that the CSPα chaperone complex increases isoproterenol-mediated signaling by stimulating GDP/GTP exchange of Gα_s_. In this report we demonstrate that in response to heat shock or treatment with the Hsp90 inhibitor geldanamycin, the J protein Hsp40 becomes a major component of the CSPα complex. Association of Hsp40 with CSPα decreases CSPα-CSPα dimerization and enhances the CSPα-induced increase in steady state GTP hydrolysis of Gα_s_. This newly identified CSPα-Hsp40 association reveals a previously undescribed coupling of J proteins. In view of the crucial importance of stress-induced chaperones in the protection against cell death, our data attribute a role for Hsp40 crosstalk with CSPα in neuroprotection.

## Introduction

In response to a range of stressful stimuli including hyperthermia and ischemia, an ancient evolutionarily conserved cellular program called the heat shock response is activated and the expression of several chaperones is induced to enhance cell survival to subsequent insults. The heat shock response also involves the translocation of several chaperones [1], [2]. Although the mechanistic basis of chaperone cytoprotection is not yet understood, the chaperone anti-apoptotic activity is thought to be due to the ability of chaperones to rid the cell of misfolded proteins. In addition to the stress-induced chaperones, many molecular chaperones are expressed constitutively and are widely held to have basic and indispensable functions in maintaining protein conformation. Constitutive and stress-induced chaperones maintain an important balance in the cell between protein refolding and protein elimination, however, while transient up-regulation of molecular chaperones is critical for cell survival, chronic up-regulation of chaperones (eg Hsp70) correlates with poor prognosis in some types of cancer [3]. Neurons are thought to be especially vulnerable to the risk of protein misfolding due to the conformational flexibility required of the cellular machinery underlying synaptic transmission. Furthermore, post-mitotic cells, such as neurons, cannot dilute aggregated proteins through cell division, leading to the potential accumulation of misfolded proteins. Huntington's, Alzheimer's, Parkinson's and Prion diseases are caused by defects in protein folding, underlining the biological importance of the problem of aberrant protein folding in neurons. In experimental models, molecular chaperones, either constitutive or stress-induced, are inhibitors of neurodegeneration (reviewed: [4]–[6]). Pharmacological modulators that directly regulate chaperone and stress-induced chaperone activity have been identified, emphasizing the potential of the heat shock response as a pharmaceutical target (Reviewed: [7]).

Cysteine string protein (CSPα) is a synaptic vesicle protein bearing a signature J domain and a cysteine rich string region that is implicated in the defense against neurodegeneration. The cytosolic proteins Hsc70 (heat shock cognate protein of 70 kDa), SGT (small glutamine rich tetratricopeptide repeat domain protein) and HIP (Hsc70 interacting protein) form a complex with CSPα which is tethered to the synaptic vesicle. Assembly of the chaperone components is thought to allow for localized activation of Hsc70, a cytosolic ATPase that couples energy from ATP hydrolysis to conformational work on target proteins [8]–[11]. The assembly of the CSPα chaperone complex is nucleotide sensitive, emphasizing the dynamic nature of the complex [8], [10], [12]. Why a specialized synaptic vesicle chaperone system evolved remains a mystery.

The existence of the heat shock response raises several questions. How do stress-induced chaperones protect synaptic transmission and prevent neurodegeneration? Is there crosstalk between stress-induced chaperones and constituitively expressed chaperones or do these chaperone machines have separate non-overlapping cellular tasks? As a first step toward testing the hypothesis that stress-induced chaperones are coupled to the CSPα chaperone system, we evaluated the components of the CSPα complex during transient expression of stress chaperones. CSPα expression levels do not increase in response to heat shock or geldanamycin treatment in neural cell lines. The previously reported 70 kDa CSPα dimer [13], [14] was reduced following heat shock. Suprisingly our findings demonstrate that the stress-induced J protein Hsp40 becomes a major component of the CSPα multimeric complex after heat shock. Following heat shock, Hsp40 expression is increased and Hsp40 localizes to the plasma membrane. Geldanamycin, like heat shock, triggers the assembly of Hsp40 with the CSPα multimeric complex. *In vitro*, the stimulatory effect of CSPα on the steady-state hydrolysis of GTP by Gα_s_ is enhanced in the presence of Hsp40. Furthermore, transient transfection of CSPα or induction of the heat shock response increased isoproterenol-stimulated phosphorylation of synapsin.

Our results suggest that the transient assembly of Hsp40 with the CSPα complex is important in the maintenance of synaptic function in the face of environmental stress, and emphasize the complexity and functional elegance of the J protein chaperone machines.

## Results

### CSPα expression is not altered in response to heat shock

**Figure 1:**
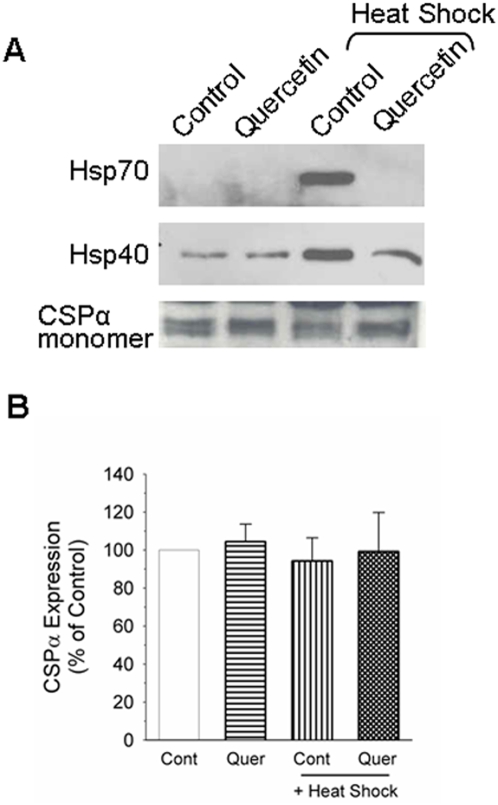
Effect of heat shock on native CSPα expression. (A) CAD cells were treated with or without 200 µM quercetin for 24 hours followed by heat shock for 40 min at 42°C and allowed to recover for 5 hours. 30 µg of protein was resolved by SDS-PAGE. CSPα, Hsp70 and Hsp40 were detected by Western analysis. (B) Quantification of CSPα. Data were derived from a total of 8 separate experiments.

To begin to test the role of CSPα in the heat shock response, the expression of CSPα and Hsp70 was examined in CAD mouse neuroblastoma cells before and after 40 min of conditioning heat shock at 42°C. CSPα is constitutively expressed in brain and exocrine/endocrine secretory tissues, however, its expression has been reported to be influenced by antidepressants [15]–[17], amphetamines [18] and diabetes [19]. The cellular mechanisms that underlie changes in CSPα expression are currently unknown. The cDNA clone for rat CSPα (594 bp open reading frame) contains 181 nucleotides of 5′ untranslated region, 1.2 kb of 3′ untranslated region and encodes a 35 kDa protein with extensive lipid modification [13]. Figure 1 shows that CSPα levels are not increased by heat shock. As expected, Hsp70 and Hsp40 were induced by heat shock and their induction was blocked by pretreatment with quercetin, an inhibitor of the heat shock response. Quercetin did not alter CSPα expression.

**Figure 2:**
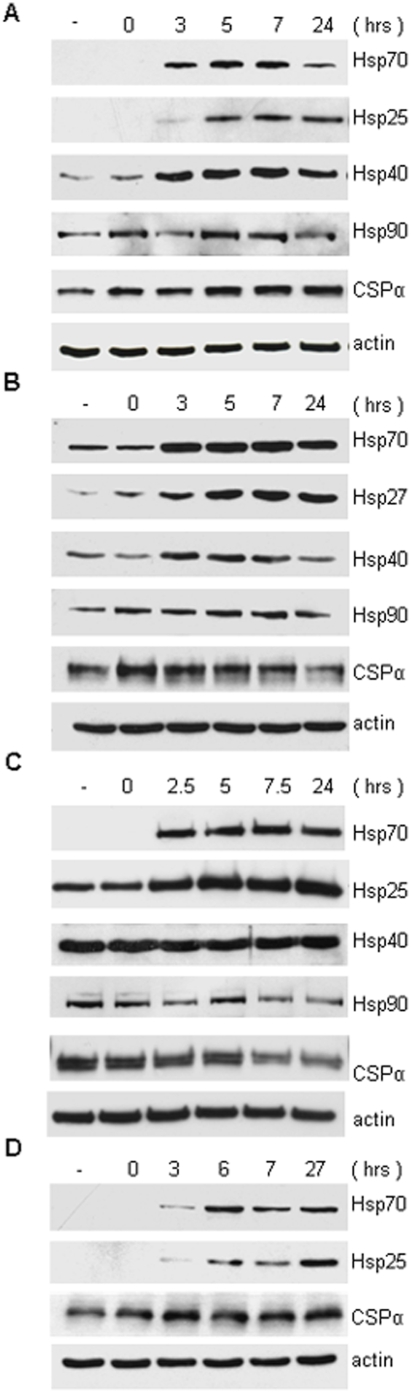
Time course of heat shock in CAD, LAN1, PC12 and differentiated CAD cells. (A) CAD, (B) LAN1 and (C) PC12 cells were heat shocked for 30 min at 42°C and allowed to recover for indicated times. (-) no heat shock. (D) CAD cells were differentiated by 36 hours serum starvation. 20 µg of protein was resolved by SDS-PAGE. Hsp70, Hsp25, Hsp27, Hsp40, Hsp90, CSPα and actin were detected by Western analysis. Data are representative of 4 separate experiments.

We then evaluated the time course of expression of select chaperones in response to heat shock. Following a conditioning stress, Hsp70 is rapidly expressed in CAD, LAN1, and PC12 cell lines, consistent with Hsp70 induction in neurons [1]. Figure 2 demonstrates that Hsp70 protein expression is clearly increased ∼3 hours after heat shock. Although Hsp70 is detected in LAN1 cells prior to the conditioning stress, the levels of Hsp70 expression were still observed to increase in response to heat shock. The time course of Hsp70 expression showed slower onset in differentiated compared to undifferentiated CAD cells, however robust Hsp70 expression was observed at ∼6 hours in differentiated CAD cells. Figure 2 shows that Hsp25/27 expression also increased in CAD, LAN1, PC12 cell lines ∼5 hours after a conditioning heat shock. Although Hsp25/27 was expressed in unstressed LAN1 and PC12 cells, further increases were observed following a conditioning heat shock. Hsp40 levels increased ∼3 hours after a conditioning heat shock in CAD and LAN1 cells, however no increase was seen in PC12 cells which already had high control levels of Hsp40 pre-heat shock. In contrast to Hsp70, Hsp40 and Hsp25/27, the expression of actin was not altered in response to heat shock demonstrating the specificity of the cellular response to a conditioning stress. Following heat shock CSPα levels are not altered in CAD cells (Figure 1), however in PC12 and LAN1 cells reduced CSPα levels were observed (Figure 2). These data are consistent with those found *in vivo* where the severity of the stress required to trigger the heat shock response is reported to vary among neural populations [6].

### Hsp70 and Hsp25/27 expression is variable among neural cell lines

**Figure 3:**
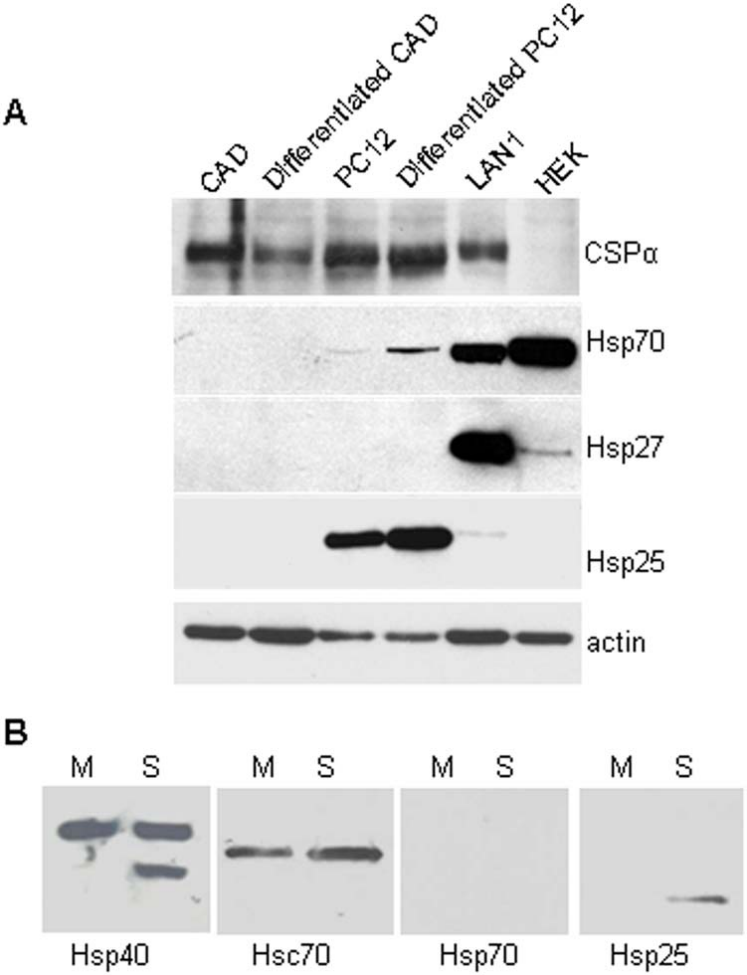
CSPα expression in CAD, PC12, LAN1, HEK cells. CAD cells were differentiated by serum withdrawal. PC12 cells were differentiated by NGF (50 ng/ml 9 days). (A) 25 µg of total cell homogenate. (B) Basal chaperone expression levels in rat brain supernatant (S) or rat brain membranes (M) were resolved by SDS-PAGE. CSPα, Hsp70, Hsp27, Hsp25 and actin (loading control) were detected by Western analysis. Data are representative of 4 separate experiments.

Next we directly compared non-heat shocked (control) CAD, differentiated CAD, PC12, differentiated PC12, LAN1 and HEK cells for CSPα expression. Figure 3A shows that CSPα was present in all cell lines except HEK cells. The cell lines were then evaluated for basal (control) levels of heat shock chaperones including Hsp70, Hsp25/27 (heat shock protein 25/27) and Hsp40. Hsp70 was not detectable in non-heat shocked (control) CAD, differentiated CAD or PC12 cells, but was present at high levels in LAN1 and HEK cells and detectable in differentiated PC12 cells. Notably, some tumor cells have been reported to constitutively express high levels of the anti-apoptotic chaperone Hsp70. Furthermore, high Hsp70 levels have been reported to correlate with poor prognosis in some types of cancer [3], [7]. In contrast, Hsp25/Hsp27 was absent from CAD cells but detectable in HEK cells and abundant in LAN1 and PC12 cells. Hsp25 and Hsp27 are homologous proteins. Hsp25 is present in rat and mouse, while Hsp27 is present in humans. Anti-Hsp25 antibody does not cross-react with Hsp27 and vice versa. Actin is shown as a loading control (Figure 3, panel 5). The basal expression of select chaperones in unstressed rat brain is shown for comparison in Figure 3B. Hsp40 and Hsc70 are abundant in both the cytosolic (S) and membrane particulate (M) fractions of rat brain while the stress-inducible Hsp70 was not detected. Hsp25 was detected in the cytosolic fraction. Taken together, these observations indicate that while CSPα was present in all neural cell lines as expected, the background expression of stress-induced chaperones varied extensively between the cell lines.

### The CSPα dimer is reduced by heat shock and increased by quercetin treatment

**Figure 4:**
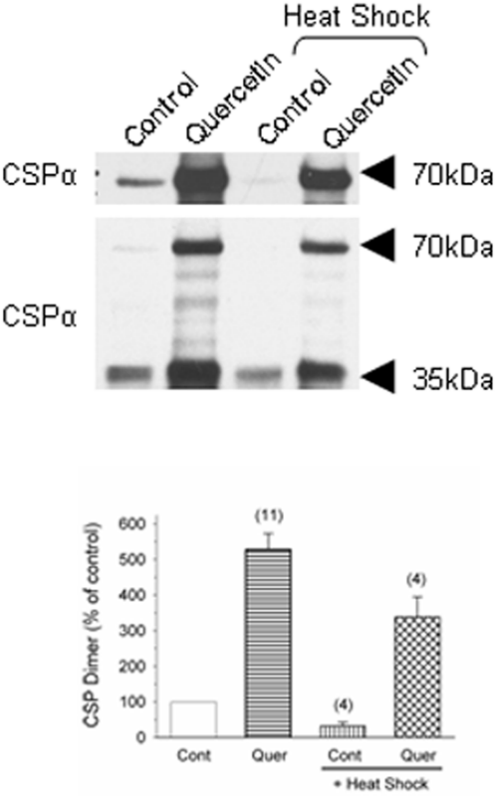
Effect of heat shock on the CSPα dimer. CAD cells were transfected with CSPα, treated as indicated with 200 µM quercetin and then heat shocked for 40 min at 42°C and allowed to recover for 5 hours. CSPα was detected by western analysis. Quantification of CSPα dimer. N values are indicated in brackets.

Although CSPα is present in neural cell lines, its expression is ∼10 fold lower (10±1.7, n = 3, data not shown) than that found in rat brain homogenates. To conduct a more detailed analysis of the CSPα chaperone complex, CSPα was examined in CAD mouse neuroblastoma cells transiently transfected with CSPα in order to bring CSPα levels up to those found in adult rat brain. After transfection a 70 kDa CSPα immunoreactive band was observed (Figure 4). 70 kDa CSPα dimers have previously been reported in rat brain [13], [20], rat hippocampus [12], rat pancreas [13], PC12 cells transiently expressing CSPα [14], [21], and HEK293 cells transiently expressing CSPα [22]. The cellular role of the CSPα dimer is not known. Figure 4 shows that in CAD cells transfected with CSPα the 70 kDa CSPα dimer is stable, SDS-resistant and maintained after incubation in sample buffer at 80°C for 10 min. Figure 4 clearly demonstrates that following 40 minutes of conditioning heat shock at 42°C, there is a decline in the CSPα dimer detected. In contrast, quercetin increases the CSPα-CSPα complex in control and heat shocked cells (Figure 4). The upper panel in Figure 4 is an overexposure of the 70 kDa CSPα dimer demonstrating its presence in control but not heat shocked CAD cells. In transfected (Figure 4) but not untransfected (Figure 1) CAD cells, quercetin was observed to increase expression levels of the CSPα monomer. Taken together, these data show that the extremely stable CSPα dimer is regulated by quercetin as well as by a conditioning heat shock.

### Hsp40 is a major component of the CSPα complex after a conditioning heat shock

Two possible scenarios could mediate the anti-apoptotic activity of stress chaperones. It is possible that (1) stress inducible chaperones have evolved to perform the same function as constitutively expressed chaperones or that (2) they carry out specialized functions to specifically cope with physiological stress. In order to distinguish between these two possibilities, we examined the association between CSPα and heat shock proteins. Glutathione-S-transferase (GST) fusion proteins consisting of CSPα_1-112_ or CSPα_1-198_ (full length) were coupled to glutathione agarose beads and used in an *in vitro* binding assay. The beads were incubated with cell homogenate, washed, and bound proteins were eluted and evaluated by Western blot. We have previously shown that CSPα_1-112_ contains two binding sites for G proteins and has guanine nucleotide exchange (GEF) activity for Gα_s_ [11]. Recombinant full length CSPα_1-198_ also contains both binding sites for G proteins, but only has GEF activity for Gα_s_ in the presence of Hsc70 and SGT (small glutamine-rich tetratricopeptide repeat domain protein). Specifically, we have shown that CSPα regulates heterotrimeric GTP binding proteins (G proteins) by preferentially targeting the inactive GDP-bound form of Gα_s_ and promoting GDP/GTP exchange which increases cAMP levels and downstream phosphorylation. *In vitro*, Hsc70 and SGT trigger a switch in CSPα from an inactive GEF to an active GEF. Therefore we examined both CSPα_1-198_ (requires Hsc70/SGT activation) and CSPα_1-112_ (active GEF) for their possible associations with stress-induced chaperones.

**Figure 5:**
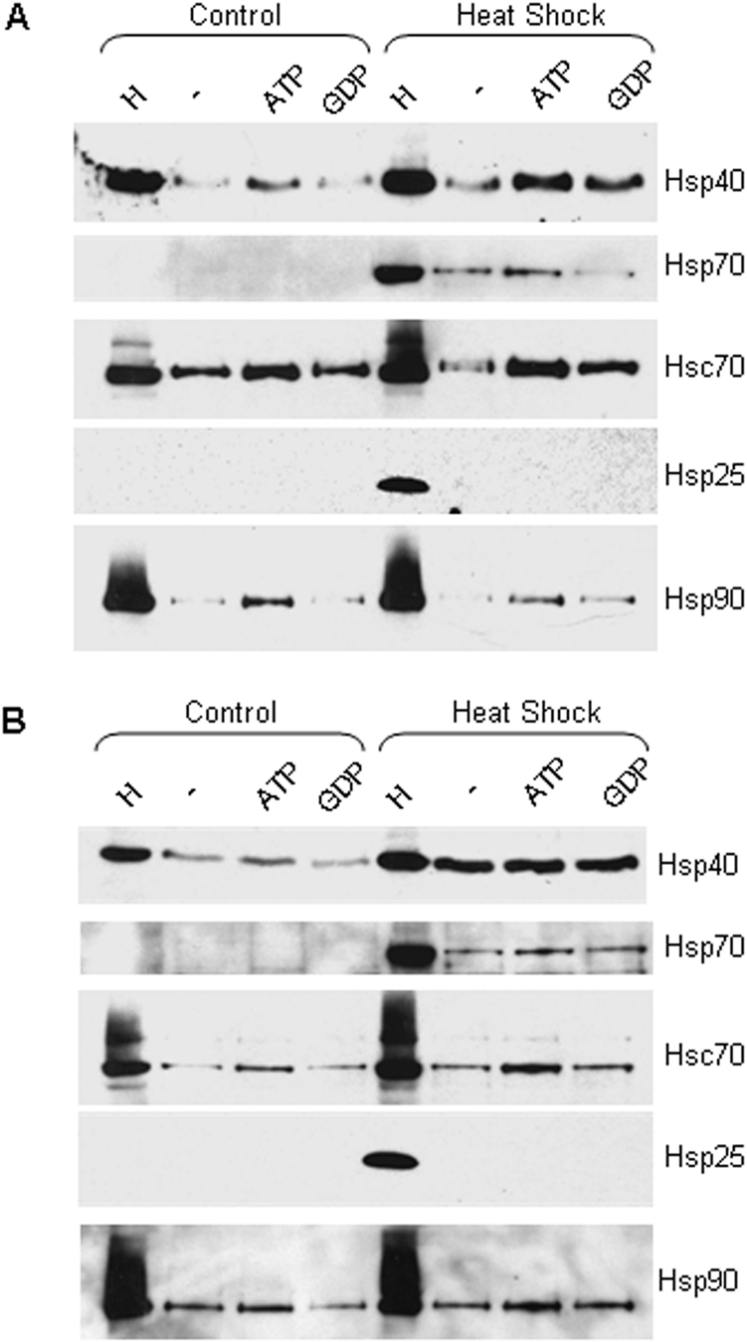
Western analysis showing the association of Hsp40 with CSPα_1-112_ and CSPα_1-198_ before and after heat shock. (A&B) CAD cells were heat shocked for 30 minutes at 42°C and allowed to recover for 5 hours. GST fusion proteins of (A) CSPα_1-112_ and (B) CSPα_1-198_ were immobilized on glutathione-sepharose and incubated in the presence of 110 µg of control or heat shocked CAD cell homogenate in the presence or absence of 2 mM ATP or GDP. The lane indicated as (H) is 40 µg of cell homogenate loaded directly on the gel. The beads were washed and bound proteins were eluted in sample buffer, fractionated by SDS-PAGE and subjected to Western blot analysis. Hsp70, Hsc70, Hsp25, Hsp40, and Hsp90 were detected by Western analysis.

Chaperones homologous to those found in the CSPα complex are induced following a preconditioning heat shock (eg. Hsp70 (70 kDa heat shock protein) and Hsp40 (40 kDa heat shock protein)). Hsc70 and Hsp70 both associate with the J domain of CSPα [8], [9], [20]. Despite the robust induction of Hsp70 expression by heat shock and its significant homology (85%) with Hsc70 [23], a CSPα/Hsc70 complex was favored over a CSPα/Hsp70 complex in CAD cell homogenates (Figure 5). After a conditioning heat shock, Hsp70 was induced and associated weakly with the GST-CSPα_1-112_ and CSPα_1-198_ complex. These data confirm the relatively weaker CSPα association and ATPase activation of Hsp70 compared to Hsc70 utilizing yeast two hybrid and ATPase assay techniques [9], [20]. The ATPase Hsp90 weakly associated with the CSPα complex. Like Hsc70, Hsp70 association was increased in the presence of ATP. In contrast, Hsp25 was not found to associate with the CSPα complex either before or after heat shock.

If Hsp40 and CSPα have evolved to perform separate functions, then it is possible that induction of Hsp40 will reduce the assembly of CSPα with Hsc70. Given the homology between the J domains of CSPα and Hsp40, one might expect elevated levels of Hsp40 to disrupt the CSPα/Hsc70 complex and favor a Hsp40/Hsc70 complex by competing with CSPα for association with Hsc70 and Hsp70. To our surprise, however, Hsp40 was found to associate robustly with both GST-CSPα_1-112_ and CSPα_1-198_ after heat shock. Figure 5 shows that Hsp40 association with CSPα_1-198_ was nucleotide independent, however association of Hsp40 with CSPα_1-112_ was greater in the presence of either ATP or GDP. These data indicate that the components of the CSPα chaperone complex are altered in response to a conditioning stress to include the cytosolic stress-induced J protein Hsp40.

**Figure 6:**
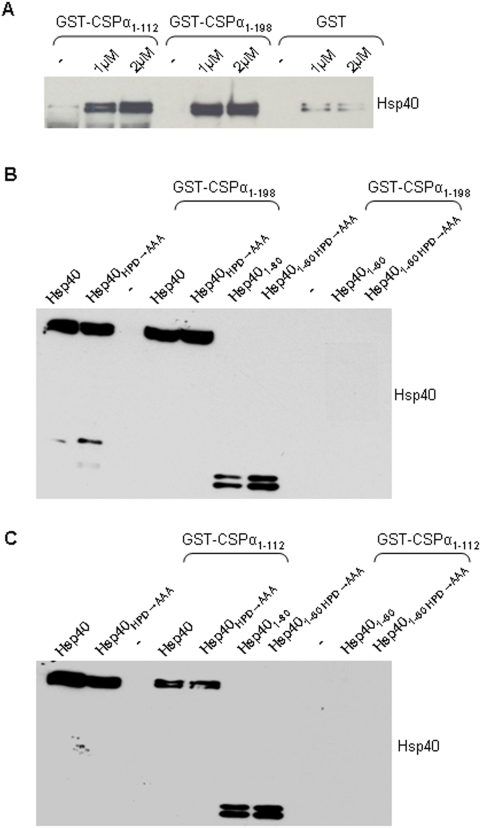
Western analysis showing association of CSPα fusion proteins with recombinant Hsp40 and Hsp40_HPD→AAA_. (A) Immobilized GST-CSPα_1-112_, GST-CSPα_1-198_, or GST alone were incubated with full length Hsp40 (i.e. amino acids 1–340), washed and bound Hsp40 was evaluated by Western blot. (B) Immobilized GST-CSPα_1-198_ was incubated with soluble, full length Hsp40_1-340_, Hsp40_1-340HPD→AAA_, Hsp40_1-80_ or Hsp40_1-80HPD→AAA_. Beads were washed, bound proteins eluted in sample buffer and subjected to Western blot analysis with anti-Hsp40 polyclonal (Assay Designs). Lanes are as indicated: (lanes 1–3 loaded directly on the gel) 0.5 µM Hsp40_1-340_, 0.5 µM Hsp40_1-340HPD→AAA_, GST-CSPα_1-198_, lanes 4&5 pull down of 1 µM Hsp40_1-340_, 1 µM Hsp40_1-340HPD→AAA_ with GST-CSPα_1-198_. (lanes 6–8 loaded directly on the gel) 0.5 µM Hsp40_1-80_, 0.5 µM Hsp40_1-80HPD→AAA_, GST-CSPα_1-198_. (lanes 9&10) pull down of 1 µM Hsp40_1-80_, 1 µM Hsp40_1-80HPD→AAA_ with GST-CSPα_1-198._. (C) As for B, however the immobilized protein was GST-CSPα_1-112_. Data are representative of at least four similar experiments.

While these data are consistent with a direct interaction between Hsp40 and CSPα, it does not permit us to rule out the possibility that CSPα/Hsp40 interact indirectly. To investigate this possibility, we examined the ability of immobilized CSPα fusion proteins to interact with soluble Hsp40. As shown in Figure 6A, immobilized GST-CSPα_1-198_ and GST-CSPα_1-112_ were able to bind purified, soluble Hsp40 in a pull down assay, indicating that there is indeed a direct interaction between CSPα and Hsp40.

To further understand the structural requirements for assembly of Hsp40 with the CSPα complex, Hsp40 deletion and point mutants were constructed and the regions of Hsp40 required for its binding to CSPα were determined. In each assay, an equal amount of fusion protein was immobilized to sepharose beads as confirmed by Ponceau S staining. The presence of Hsp40 was analyzed by Western blotting. Hsp40 and CSPα belong to a large and diverse protein family [24]. Each member has a conserved J domain that functions to stimulate Hsc70/Hsp70 ATPase (eg CSPα stimulates Hsc70 ATPase [8]). There is no functional one to one correspondence between members of the J protein family and members of the Hsp70 family. J domains are a ∼70 amino acid region of homology comprised of four α helices with a highly conserved tripeptide of histidine, proline and aspartic acid (HPD motif) located between helices II and III. The structures of the J domain of CSPα (mouse) and Hsp40 (human) have been determined from nuclear magnetic resonance studies [25]. Figure S1 shows the comparison of the amino acid sequence between rat CSPα and Hsp40. The J domain (magenta) and cysteine string region (red) of CSPα and the J domain and the DnaJ C-terminal domain (cyan) of Hsp40 are indicated. CSPα and Hsp40 show only 16% identity over their entire amino acid sequences, but 51% identity within the J domains. Secondary structure predictions for helix 1 show a weak “β sheet” (the probability is 0.4 sheet compared to 0.3 helix; 1 is high, 0 is low) for position 5–8 a.a. of Hsp40_Rat and 17–20 a.a. of CSPα_Rat, while predictions are stronger for helices 2, 3 and 4 (probability is 1, 1 and 0.7 respectively). Mutation of the highly conserved HPD tripeptide of Hsp40 (Hsp40_HPD→AAA_) did not abolish binding to CSPα, indicating that CSPα/Hsp40 association is not dependent on this conserved motif. Furthermore, Hsp40_1-80_ as well as Hsp40_1-80HPD→AAA_ were not found to associate with CSPα_1-198_ or CSPα_1-112_ (Figure 6B&C). These data therefore define Hsp40 residues 81–340 as important for binding to CSPα.

**Figure 7:**
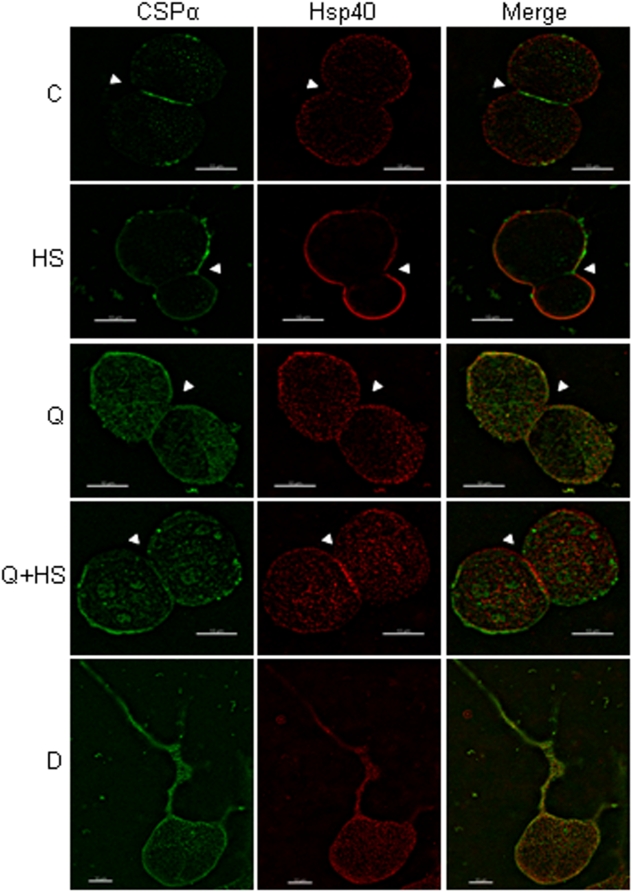
Localization of Hsp40 and CSPα. Immunolocalization of CSPα (green) and Hsp40 (red) in CAD cells transiently expressing myc-CSPα under indicated conditions (control (C), heat shock (HS), 100 µM quercetin (Q), quercetin and heat shock (Q+HS) or differentiated control (D)). Scale bar = 10 µm. Deconvolution was performed with Imaris 4.1. The merged fluorescent images are shown in the right-hand panel.

To understand better the nature of the CSPα/Hsp40 association, we evaluated the distribution of CSPα and Hsp40 in CAD cells transiently expressing myc-CSPα (Figure 7). In control cells (C), CSPα was observed to be concentrated at the cell-cell contact sites (indicated with an arrow) and, in contrast, Hsp40 was broadly expressed. Following heat shock (H.S.) we found CSPα and Hsp40 to be present primarily in the plasma membrane however reduced at the cell-cell contacts, indicating that Hsp40 as well as CSPα was relocalized. These observations are consistent with our biochemical data (Figures 5&6) demonstrating a CSPα∶Hsp40 association following heat shock. Panel 4 clearly demonstrates that quercetin blocked the heat shock general redistribution of Hsp40 to the plasma membrane. Intracellular localization of CSPα and Hsp40 was increased in cells treated with quercetin (Q) as well as quercetin followed by heat shock (Q+HS). To our surprise, Hsp40 but not CSPα localized to cell-cell contacts following the quercetin/heat shock. Plasma membrane co-localization of CSPα and Hsp40 in single differentiated cells is shown in yellow in the right hand panel (panel 5). Taken together, these biochemical and histochemical data demonstrate that following heat shock, cellular levels of Hsp40 are increased and both Hsp40 and CSPα undergo redistribution. These data indicate that changes in the expression levels of Hsp40 as well as changes in the cellular localization of Hsp40 and CSPα facilitate the assembly of Hsp40 with the CSPα chaperone complex.

### Hsp40 is a major component of the CSPα complex after Geldanamycin treatment

Next we evaluated a series of agents to see if they altered either CSPα levels or the components of the CSPα complex (Figure S2A). The Hsp90 ATPase inhibitors geldanamycin, 17-AAG and novobiocin robustly induced the expression of Hsp70 in PC12 cells. Lithium ions have been reported to enhance CSPα expression [26] and we have previously shown that CSPα increased the response to the β2 adrenergic agonist isoproterenol [11]. No Hsp70 was detected in PC12 cells treated with isoproterenol, LiCl or quercetin. In contrast to Hsp70 and Hsp25, the expression of actin was not altered in response to geldanamycin or 17AAG demonstrating the specificity of the cellular response to the Hsp90 inhibitors. Following geldanamycin treatment of PC12 cells, Hsp70 expression is detected as early as 5 hours and continues to increase (Figure S2B). To our surprise, geldanamycin did not trigger the expression of Hsp70 but did induce Hsp40 expression without increasing CSPα expression in CAD mouse neuroblastoma cells (Figures S2C&D). The molecular events that underlie the difference between geldanamycin-induced and heat shock-induced Hsp70 expression in CAD cells remains to be established, however, it provided us the opportunity to investigate the CSPα/Hsp40 association in the absence of Hsp70.

**Figure 8:**
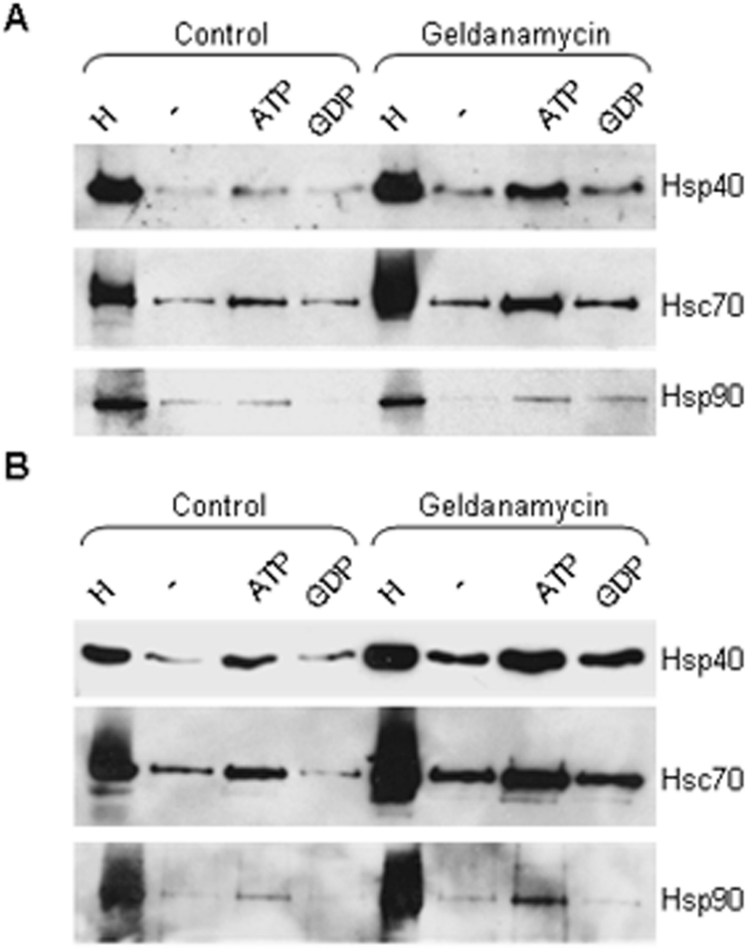
Western analysis showing the association of chaperones with CSPα_1-112_ and CSPα_1-198_ before and after geldanamycin (2 µM) treatment. Fusion proteins of (A) CSPα_1-112_ and (B) CSPα_1-198_ were immobilized in sepharose and incubated in the presence of 110 µg of control or geldanamycin-treated CAD cells in the presence or absence of 2 mM ATP or GDP. The beads were washed and bound proteins were eluted in sample buffer, fractionated by SDS-PAGE. Hsc70, Hsp40, and Hsp90 were detected by Western analysis.

We then examined the CSPα complex in geldanamycin treated CAD cells. CSPα_1-112_ or CSPα_1-198_ fusion proteins were coupled to glutathione agarose beads and used in an *in vitro* binding assay. Following geldanamycin treatment, Hsp40-CSPα_1-112_ and Hsp40-CSPα_1-198_ complexes were abundant in the presence of ATP (Figure 8), similiar to that found after heat shock (Figure 5). Again, Hsp90 was observed to associate weakly with the CSPα complex. Thus the CSPα chaperone complex is dynamic, undergoing changes in components in the post-stress response and in response to Hsp90 inhibitors. Taken together, our results indicate that as the cellular levels of Hsp40 rise in response to a conditioning stress or after treatment with Hsp90 inhibitors, the CSPα complex becomes an Hsp40-CSPα complex.

### Hsp40 promotes CSPα's GEF activity in vitro and synapsin phosphorylation in CAD neuroblastoma cells

**Figure 9:**
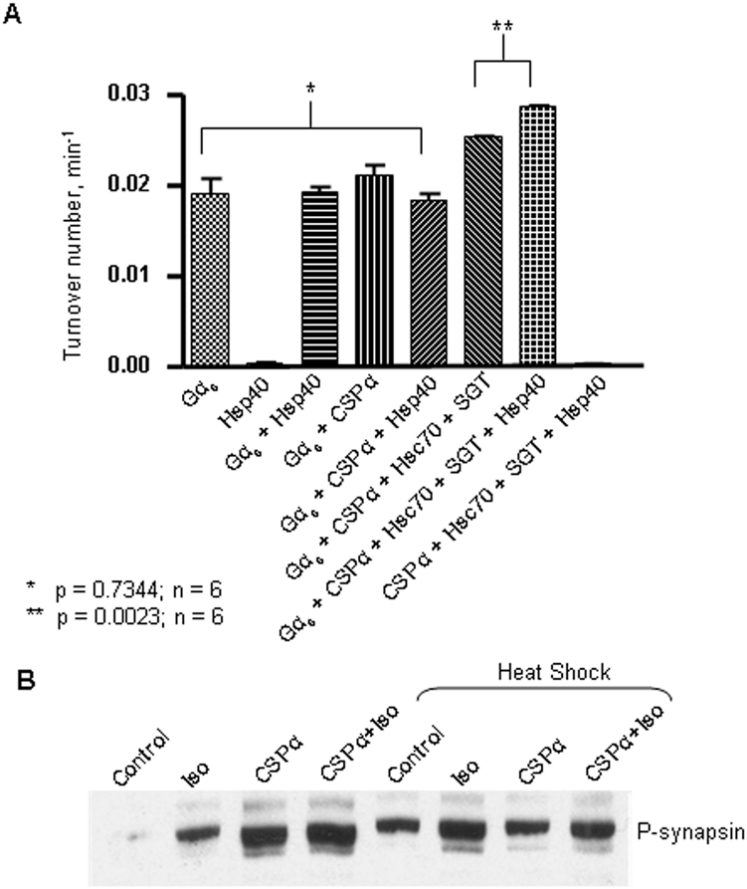
Hsp40 increases the CSPα stimulation of GTPase activity of Gα_s_. (A) Soluble full length Hsp40, CSPα, SGT and Hsc70 were mixed with γ-[^32^P]GTP (1 μCi) prior to the addition of 0.3 µM Gα_s_. The amount of released ^32^P was measured as counts/min after incubation of the reaction mixture for 30 min at 25°. p values for two-tailed unpaired t test (95% confidence interval) were 0.7344 (*) and 0.0023 (**), respectively. n = 6. (B) Differentiated CAD cells were transfected with CSPα and heat shocked at 42°C for 40 mins. Cells were allowed to recover 5 hrs prior to treatment with 50 µM isoproterenol+50 µM IBMX for 15 min where indicated. Western analysis was carried out and the membrane probed with anti-phospho-synapsin antibody in TBS-BSA-Tween 20. For phosphosynapsin the pixel values from left to right were (control)132, (Iso)2422, (CSPα)7528, (CSPα+Iso)10146, (control&heat shock)3543, (Iso+heat shock)8910, (CSPα+heat shock) 3414, (CSPα+Iso+heat shock) 5206.

Next we evaluated Hsp40's effect on the steady state hydrolysis of GTP by Gα_s_. We have previously demonstrated that the stimulation of GTPase activity by Gα_s_ by CSPα requires Hsc70 and SGT [11]. Figure 9A shows that Hsp40 enhanced the CSPα-Hsc70-SGT-stimulated increase in GTP hydrolysis by Gα_s_. GTP hydrolysis by Gα_s_ was not altered in the presence of Hsp40 alone, indicating that Hsp40 does not effect the hydrolysis of GTP by Gα_s_ alone. Furthermore, Hsp40 does not hydrolyze GTP. To gain further insight into the transient association of Hsp40 with the CSPα chaperone complex, we evaluated the activation of cellular mechanisms downstream of G proteins after heat shock. Phosphorylation of synapsin in CAD cells serves as an independent readout of cellular signaling through Gα_s_. Figure 9B shows that isoproterenol-induced phosphorylation of synapsin in CAD cells was increased after heat shock (3.7 fold) or transfection with myc-tagged CSPα (4.2 fold). This is consistent with previous work showing that expression of CSPα in HEK cells increased isoproterenol-stimulated cAMP levels [11] as well as G protein inhibition of N-type calcium channels [12]. Moreover, we have recently reported that the expression of CSPα in CAD cells increases isoproterenol-mediated phosphorylation of the transcription factor CREB [27]. These results show that the heat shock response modulates signaling through Gα_s_ pathways.

## Discussion

In this study, we demonstrate for the first time that the stress-induced J protein, Hsp40, physically couples with the constitutive CSPα chaperone complex. CSPα is a synaptic vesicle J protein that is essential for periods of extended neurotransmission and is implicated in the defense against neurodegeneration [28]–[30]. CSPα is constitutively expressed on synaptic vesicles and is thought to tether Hsc70 for conformational work at the synaptic vesicle site. Hsp40 is a cytosolic J protein that is rapidly and transiently induced in response to stress, including, but not exclusive to, heat shock and geldanamycin treatment. Stress-induced expression of several chaperones, including Hsp40, is widely thought to protect cells from the deleterious effects of subsequent stress. Interference with the heat shock response would be expected to have enormous cellular consequences and reduce cell survival. It is fully anticipated that transient expression of stress-induced chaperones *in vivo* occurs routinely in neurons to cope with the stresses rendered by various insults. Intriguingly, some neurons have a particularly higher threshold for the induction of the heat shock response compared with other neurons [6]. In this study, we provide evidence that Hsp40 specifically and directly associates with the constitutive CSPα synaptic vesicle chaperone complex following either a conditioning heat shock or geldanamycin treatment. Following heat shock, Hsp40 expression is increased and both CSPα and Hsp40 undergo redistribution. The association of Hsp40 with CSPα correlates with reduced CSPα dimerization, translocation of Hsp40 to the plasma membrane and enhanced CSPα-mediated augmentation of steady state GTP hydrolysis. Association with CSPα is mediated via C terminal binding sites of Hsp40 and does not involve the J domain. Since neurotransmitter release relies on complex interactions between multiple cellular components, we speculate that the cross-talk between CSPα chaperone machinery and the stress-induced Hsp40 is important in maintaining functionally competent synapses.

Whether stress-induced chaperones typically perform independent cellular tasks or are coupled to constitutive chaperone machines, such as CSPα, is a current biological question. The notion that Hsp40 contributes to the folding activity of the CSPα chaperone complex after a conditioning stress is consistent with the neurodegeneration observed in CSPα deletion models. Deletion of the CSPα gene severely impairs central and presynaptic transmission in *Drosophila melanogaster* [30]–[32]. The *Drosophila* CSPα null mutants exhibit temperature sensitive paralysis and die as larvae or within days of adulthood [30]. Deletion of CSPα in mice causes blindness followed by progressive motor and sensorial impairment and neurodegeneration with no survival beyond 4 months [29], [33], [34]. Thus, these reports indicate that CSPα is important for the long term functional integrity of the synaptic machinery. It is possible, given the data presented here that the prevention of presynaptic neurodegeneration by the synaptic vesicle CSPα chaperone complex involves a transient interaction with the highly conserved stress induced protein Hsp40.

Candidates for the protein substrate(s) of the CSPα/Hsc70 system include: G proteins (heterotrimeric GTP binding proteins) [11], [12], [35], voltage sensitive calcium channels [36], [37] and SNAREs (soluble N ethylmaleimide-sensitive factor attachment protein receptors) [28], [38], [39]. Furthermore, CSPα has been shown to be critical for the normal calcium sensitivity of synaptic exocytosis [40], [41]. Several misfolded proteins such as huntingtin [42]–[46], PrP^C^ [47], α-synuclein [48] and ataxin 1&3 [49], [50] are known substrates for the Hsp40/Hsc70 system. It is tempting to speculate that the assembly of Hsp40 with the CSPα chaperone complex may expand the client protein(s) targeted by the synaptic vesicle CSPα complex by shifting chaperone activity from dedicated to indiscriminant.

In conclusion, our combined findings suggest a model in which heat shock alters the composition of the CSPα complex to include Hsp40. Understanding the composition of the CSPα chaperone complex, either basal or following a conditioning stress, is crucial to understanding the physiological role(s) of CSPα. Data presented here show that *in vitro*, Hsp40 increased the CSPα-induced increase in steady state GTP hydrolysis of Gα_s_. Furthermore, in CAD cells, induction of the heat shock response decreased CSPα dimerization and increased isoproterenol-stimulated phosphorylation of synapsin. In view of the crucial importance of stress-induced chaperones in protection against cell death, our data attribute a key role to the association of Hsp40 with the CSPα chaperone complex in neuroprotection.

## Materials and Methods

### Reagents and Chemicals

Anti-CSPα polyclonal was prepared as described previously [13]. Anti-Hsp70 mouse monoclonal, anti-Hsp40 rabbit polyclonal, anti-Hsp90 rat monoclonal, anti-Hsp25 rabbit polyclonal, anti-Hsp27 mouse monoclonal were from Assay Designs. Anti-phosphosynapsin and anti-synapsin rabbit polyclonal antibodies were from Cell Signaling Technology. Anti-c-myc mouse monoclonal was from Clonetech. Anti-actin mouse monoclonal, anti-Hsp70/Hsc70 mouse monoclonal, quercetin and isoproterenol were from Sigma. Geldanamycin was from Calbiochem. 17AAG was from Invitrogen. Novobiocin was from EMO Biosciences Inc.

*CAD mouse neuroblastoma cells* were seeded into 6 well plates and grown in DMEM/F12 medium supplemented with 10% fetal bovine serum and 1% Penicillin/streptomycin. For differentiation cells were grown in Opti-MEM for 60 hrs. *LAN1 human neuroblastoma cells* were seeded into 6 well plates and grown in RPMI medium supplemented with 10% fetal bovine serum and 1% Penicillin/streptomycin. *PC12 cells* were obtained from ATCC. PC12 cells were grown in Dulbecco's modified Eagle's Medium supplemented with 10% heat-inactivated horse serum and 5% fetal calf serum. For differentiation PC12 cells were treated with 50 ng/ml mouse NGF (R&D systems) for 9 days. *Human embryonic kidney tsa-201 (HEK)* cells were grown in Dulbecco's modified Eagle's medium supplemented with 10% fetal bovine serum. Cells were lysed in 40 mM Tris (pH 7.4), 150 mM NaCl, 2 mM EDTA, 1 mM EGTA, 1 mM Na_3_VO_4_, 0.1% SDS, 1% Tx100, 0.5 mM PMSF and protease inhibitor (Sigma) end-over-end at 4°C for 1 hr. Lysates were centrifuged at 15000×g for 5 min at 4°C and the supernatant was collected. Protein concentration was determined using a Bradford reagent (BioRad).

### Transient transfection of CAD cells

CAD cells were washed in PBS and transiently transfected with 0.5 ug myc-tagged rat CSPα_1-198_ DNA using Lipofectamine-2000 (Invitrogen) in Opti-MEM, and maintained in culture for 24 hrs prior to heat shock or drug treatment.

### Cell lines and lysate preparation

Whole rat brains were homogenized in 20 mM Tris-HCl buffer (pH 7.4), 2 mM MgSO_4_, 1 mM PMSF and EDTA-free inhibitor cocktail as previously described [51]. The homogenate was centrifuged at 100,000×g for 1 h at 4°C. The resultant soluble fraction was removed and designated the soluble cytosolic fraction (S). The remaining pellet was solubilized in homogenizing buffer containing 1% (w/v) n-dodecyl-β-D-maltoside (Calbiochem) for 60 min at 4°C. Following centrifugation at 100,000×g for 1 hr at 4°C, the resulting supernatant constituted the detergent-solubilized membrane particulate fraction (P). All procedures were carried out in strict accordance with a protocol approved by the University of Calgary Animal Care Committee.

### Preparation of fusion proteins

Glutathione-S-transferase (GST) fusion proteins GST-Hsp40, GST-CSPα_1-198_, GST-CSPα_1-82_ and GST-Hsc70 were prepared by sub-cloning PCR products into the bacterial expression plasmids pGEX-KG or pGEX-4T, as previously described [13], [51]. Hsp40_HPD→AAA_, Hsp40_1-80_, Hsp40_1-80__HPD→AAA_ mutants were prepared by subcloning restriction or PCR fragments into the bacterial expression plasmid pGEX-KG. Following sequence verification, DNA was transformed into AB1899 or DH5α strain of *Escherichia coli*. Expression of GST fusion proteins was induced with 100 µM isopropyl-β-D-thiogalactosidase (IPTG) for 5 hours at 37°C. Bacteria were suspended in PBS, 0.05% (v/v) Tween 20, 2 mM EDTA, 0.5 mM PMSF and 0.1% (v/v) β-mercaptoethanol and lysed by two passages through a French press (Spectronics Instruments Inc.) GST fusion proteins were recovered by binding to glutathione-sepharose beads (GE Healthcare Biosciences). Beads were suspended as a 50% (v/v) slurry in 20 mM MOPS, 4.5 mM Mg acetate, 150 mM KCl, and 0.2% (v/v) Triton X-100. The concentrations of recombinant GST fusion proteins were estimated by Coomassie blue staining of SDS-polyacrylamide gels using bovine serum albumin (BSA) as a standard. The recombinant portion of the fusion protein was eluted from the beads with 50 mM Tris (pH 7.5), 150 mM NaCl, 2.5 mM CaCl_2_, 0.1% β-mercaptoethanol and 3 µg/ml thrombin.

### In vitro ‘Pull-down’ Assays

For the ‘pull down assays’, cells were lysed end-over-end in 20 mM Tris (pH 7.4), 130 mM NaCl, 2 mM MgSO_4_, 2 mM NaVO_4_, 1% n-dodecyl-β-D-maltoside, 1 mM PMSF and protease inhibitor (Sigma) for 60 min at 4°C. Lysates were centrifuged at 20,000×g for 30 minutes and the total detergent-solubilized cell lysate (supernatant) was collected. Protein concentration was determined using a Bradford reagent (BioRad). The detergent-solubilized cell lysates were incubated with bead-immobilized GST-tagged proteins in 20 mM MOPS, 4.5 mM MgAcetate, 150 mM KCl, 0.5% Tx100, and 2 mM ATP or 2 mM GDP in a final volume of 400 µl, for 1 hour at 37°C. Beads were washed twice with 200 µl of ice cold 20 mM MOPS, 4.5 mM MgAcetate, 150 mM KCl, 0.2% Tx100. Bound proteins were eluted in Laemmli sample buffer, fractionated by SDS-PAGE and analyzed by Western blotting.

### Immunoblotting

Proteins were transferred from polyacrylamide gels to nitrocellulose (0.45 µm) in 20 mM Tris, 150 mM glycine, and 12% methanol. Membranes were blocked with 4% milk solution (prepared in PBS with 0.1% Tween 20) and incubated with primary antibody for 2 hours at room temperature or overnight at 4°C. The membranes were washed in blocking solution and incubated with horseradish peroxidase-coupled secondary antibody. The signal was developed using West Pico Pierce reagent (Pierce Biotechnology, Inc.) and exposed to Kodak film.

*Sequence analysis* CSPα from *Rattus norvegicus* (accession number: NP_077075.1) and Hsp40 from *Rattus norvegicus* (accession number: EDL92267.1) were evaluated for homology between J domains. Alignments of sequences were obtained using CLUSTAL-W with default settings in place and EMBOSS pairwise global alignment using an implementation of the Needleman-Wunsch algorithm [52]. Domains were identified with InterProScan and secondary structure prediction was carried out using PredictProtein [53].

### Immunofluorescence

CAD cells were grown on coverslips coated with glycerol in DMEM/F12 or Opti-MEM for differentiation. Cells were washed in PBS, fixed in 2% paraformaldehyde for 10 min, permeabilized in ice cold methanol for 10 min and rinsed with PBS. Cells were blocked in a 3% BSA, 0.05% Tween 20 solution in PBS for 30 min. Incubations of cells with primary antibodies were carried out sequentially overnight at 4°C and 1 hr at room temperature. Following incubation with primary antibody, cells were washed 3×with PBS and incubated with either goat anti-mouse conjugated to Alexa 488 or sheep anti-rabbit conjugated to Cy3 secondary antibody in the blocking solution for 1 hour at room temperature. Following secondary antibody incubation, cells were washed 3×with PBS, mounted onto glass slides with DABCO (Sigma) and photographed with a Leica confocal microscope. The fluorophores Alexa 488 and Cy3 were excited at 470 nm and 535 nm respectively, and images were collected at 525 nm and 610 nm.

### GTPase assay

Steady-state GTPase reactions were performed at 25°C in the presence of Gα_s_ (0.3 µM), Gβ_1_γ_1_ (0.6 µM), and the absence or presence of 0.3 µM Hsp40, CSPα, Hsc70 and SGT [54]. Proteins were mixed with 10 µM [γ-^32^P]GTP (1μCi) in a final volume of 50 µl of 20 mM Tris-HCl (pH 8.0) buffer containing 130 mM NaCl and 10 mM MgSO_4_ and the reaction was started by the addition of Gα_s_. Aliquots (20 µl) were withdrawn after 30 min and transferred to 100 µl of 7% (v/v) perchloric acid. Nucleotides were precipitated with 700 µl of 10% (w/v) charcoal suspension in phosphate-buffered saline and free [^32^P_i_] was measured with liquid scintillation counting. Results were fit with linear regression.

## Supporting Information

Figure S1 Comparison of the amino acid sequences and predicted secondary structures of Rat CSPα and Rat Hsp40. (A) The locations of J domain and DnaJ C-terminal are highlighted in magenta and cyan respectively; the cysteine repeat region of CSPα_Rat is in red background. Alignments of sequences were obtained using ClustalW with default settings in place. (B) InterProScan and PredictProtein were used to identify the domains and secondary structures. Scale bar marks the length measured by amino acids.
(1.61 MB TIF)

Figure S2 Western analysis showing the expression of CSPα, Hsp40 and Hsp70 in PC12 and CAD cells treated with the indicated agents. (A) undifferentiated PC12 cells. PC12 cell cultures were treated with 200 µM quercetin, 500 nM 17-AAG, 2 µM geldanamycin, 50 µM isoproterenol, 50 ng/ml NGF, 50 mM ethanol, 0.1% v/v DMSO, 1 mM LiCl or 200 µM novobiocin for 4 days. (B) Time course of geldanamycin (2 µM) response in PC12 cells. (C) Time course of geldanamycin (2 µM) response in CAD cells. Hsp70, Hsp40, Hsp90, CSPα, Hsp25 and actin were detected by Western analysis. (D) CAD cells were treated with geldanamycin (1 µM) for 24 hrs and probed for CSPα expression. Quantification of CSPα is shown. Data were derived from a total of 7 separate experiments.
(1.09 MB TIF)
