# Supplementary figures and images for: Hsp40 Couples with the CSPα Chaperone Complex upon Induction of the Heat Shock Response
Source: PLoS One. 2009 Feb 26;4(2):e4595. doi: 10.1371/journal.pone.0004595 (PMC2643527; doi:10.1371/journal.pone.0004595)

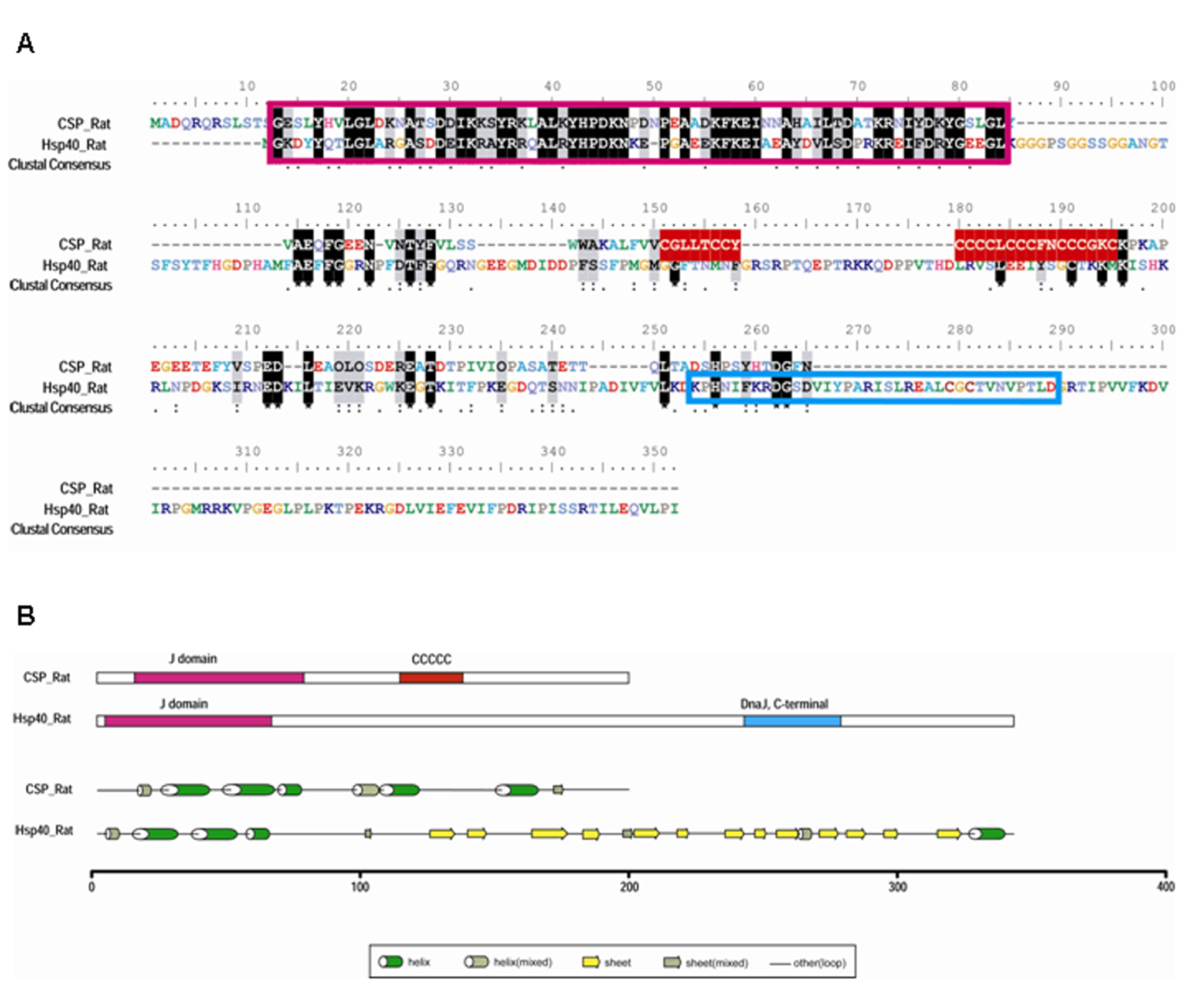

Supplement: Figure S1 — Comparison of the amino acid sequences and predicted secondary structures of Rat CSPα and Rat Hsp40. (A) The locations of J domain and DnaJ C-terminal are highlighted in magenta and cyan respectively; the cysteine repeat region of CSPα_Rat is in red background. Alignments of sequences were obtained using ClustalW with default settings in place. (B) InterProScan and PredictProtein were used to identify the domains and secondary structures. Scale bar marks the length measured by amino acids. (1.61 MB TIF) [file pone.0004595.s001.tif]

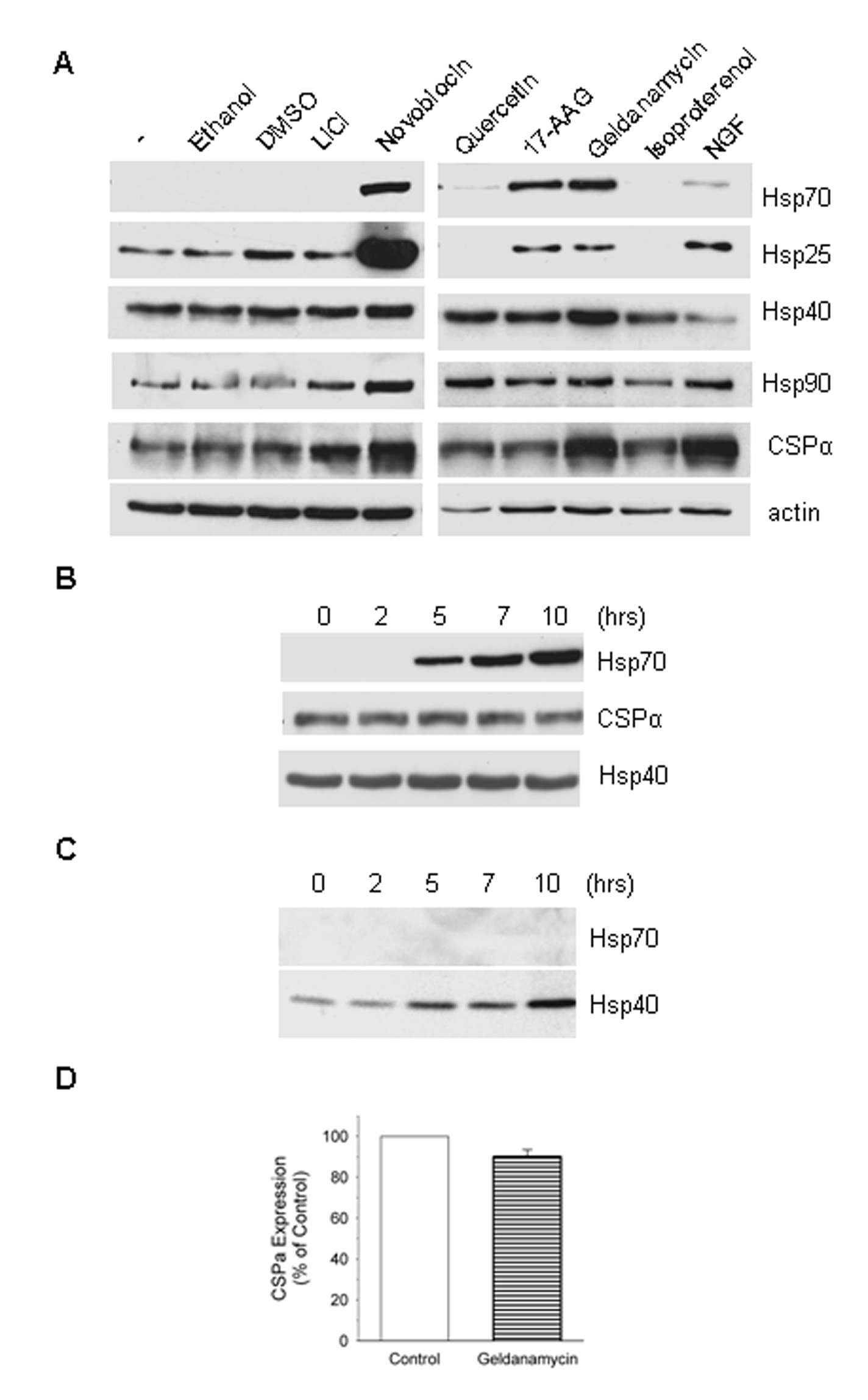

Supplement: Figure S2 — Western analysis showing the expression of CSPα, Hsp40 and Hsp70 in PC12 and CAD cells treated with the indicated agents. (A) undifferentiated PC12 cells. PC12 cell cultures were treated with 200 µM quercetin, 500 nM 17-AAG, 2 µM geldanamycin, 50 µM isoproterenol, 50 ng/ml NGF, 50 mM ethanol, 0.1% v/v DMSO, 1 mM LiCl or 200 µM novobiocin for 4 days. (B) Time course of geldanamycin (2 µM) response in PC12 cells. (C) Time course of geldanamycin (2 µM) response in CAD cells. Hsp70, Hsp40, Hsp90, CSPα, Hsp25 and actin were detected by Western analysis. (D) CAD cells were treated with geldanamycin (1 µM) for 24 hrs and probed for CSPα expression. Quantification of CSPα is shown. Data were derived from a total of 7 separate experiments. (1.09 MB TIF) [file pone.0004595.s002.tif]
